# Cross-cultural adaptatiion and validation of the stroke specific quality of life 2.0 scale into Hausa language

**DOI:** 10.1186/s41687-018-0082-1

**Published:** 2018-12-20

**Authors:** Marufat O. Odetunde, Aderonke O. Akinpelu, Adesola C. Odole

**Affiliations:** 1 Department of Physiotherapy, Obafemi Awolowo University Teaching Hospital, Ile-Ife, Nigeria; 2 Department of Physiotherapy, College of Medicine, University of Ibadan, University College Hospital, Ibadan, Nigeria

**Keywords:** Cross-cultural adaptation, Hausa, SS-QoL 2.0, WHOQoL-BREF, Validity, Reliability

## Abstract

**Background:**

The Stroke Specific Quality of Life 2.0 (SS-QoL 2.0) is a widely used scale that has been cross-culturally adapted to many languages including Yoruba, one of the three major Nigerian languages. Non-availability of SS-QoL 2.0 in Hausa, the indigenous language of Northern Nigeria has restricted its use in Hausa stroke-survivors (SSV). This study was aimed at cross-culturally adapting SS-QoL 2.0 to Hausa and assessing validity and reliability of the Hausa version. The English version of SS-QoL 2.0 was cross-culturally adapted to Hausa following the American Association of Orthopaedic Surgeons’ guideline. A final Hausa version (FHV) was produced through forward and back-translations, expert committee review, pretesting and cognitive debriefing interview. The FHV was investigated for test-retest reliability, internal consistency, convergent, construct and known-group validity on 86 consenting Hausa SSV. Hausa version of WHOQoL-BREF was used to assess convergent validity (*n* = 57) while English versions of SS-QoL was used to assess construct validity (*n* = 51) of FHV. The FHV was re-administered on 53 of the participants at 7-day interval to assess test-retest reliability. Each scale was administered in random order to eliminate bias. Data were analysed using Spearman correlation, Cronbach’s alpha, Intra-class Correlation Coefficient (ICC), Independent t-test and One-way ANOVA at *p* < 0.05.

**Results:**

The SS-QoL 2.0 was successfully cross-culturally adapted to Hausa. Participants’ mean overall score on SS-QoL 2.0 (145.30 ± 39.78) did not differ significantly from that of FHV (150.41 ± 40.45) *p* = 0.28. The mean domains score did not differ significantly except in self-care and work domains. There were weak to good correlations for 6 out of 8 similar domains on Hausa versions of SS-QoL and WHOQoL-BREF (*r* = 0.21–0.61; *p* = 0.001–0.006); and good to excellent correlations between Hausa and English versions of SS-QoL (*r* = 0.70–0.92; p = 0.001). The FHV showed high to excellent test-retest reliability (ICC = 0.86–0.99) and acceptable to excellent internal consistency (Cronbach’s α = 0.71–0.90). No significant gender differences were demonstrated for any domains of FHV and for most domains across age groups.

**Conclusion:**

The FHV is valid and reliable. The scale is recommended for assessing health-related quality of life among Hausa stroke survivors.

**Electronic supplementary material:**

The online version of this article (10.1186/s41687-018-0082-1) contains supplementary material, which is available to authorized users.

## Background

Stroke is the leading cause of adult long-term disability and death in the world [1] with a worldwide population prevalence of 0.5% [2]. Two thirds of all deaths related to stroke worldwide occur in low and middle-income countries [3]. Continued advances in medical interventions have increased the survival rate of patients who suffer strokes which has in turn increased the number of stroke survivors with chronic disability. Out of the people who suffer from a stroke, 30% die, 30% are left functionally disabled and 40% have a successful recovery with minor to no disabilities [4]. Over 50% of the stroke survivors return to their homes after hospital discharge and the impacts of stroke are considerable on them and their informal caregivers [5]. They have to deal with the long term consequences of stroke, which were seen in physical, psychological and social areas of functioning [4].

Stroke survivors have impaired or decreased quality of life (QoL) on long term basis even among those who have no post-stroke disability [6, 7]. With ongoing rehabilitation, however, improvements in functional status are possible [8] and contribute to improved QoL for stroke survivors. Hence, significant interest has arisen in health-related quality of life (HRQoL) as a tool to assess changes in patient health throughout his lifetime. Assessment of outcomes of stroke rehabilitation should therefore include disability and QoL scales to assess the domains which are directly impacted by the disease [9].

Cross-cultural adaptation encompasses a process that looks at both translation and cultural adaptation issues in the process of preparing a questionnaire for use in another setting [10] and ensures the attainment of semantic, idiomatic, experiential and conceptual equivalence between the source and the target questionnaires. It is the deliberate modification of some features of a questionnaire to better fit a particular target population [11]. It is inappropriate to simply translate and use a questionnaire in another linguistic context [12, 13]. Studies may have a comprehensive linguistic translation process, but this still do not ensure construct validity and reliability [10, 13]. Most questionnaires that clinicians use were originally developed in the English language. Cross-cultural adaptation of existing English language questionnaires would enable comparisons of different populations and permit the exchange of information across cultural and linguistic barriers [14].

Patient-reported outcome measures have been used to supplement clinician-based outcome measures [15]. This is because information from the patients’ perspective on the consequences of disease and the therapeutic benefits is considered critical in the evaluation of health care. Various specific instruments for assessing post-stroke HRQoL assess domains relevant to stroke, such as vision or language. These, however, are not available in all languages. The Stroke-Specific Quality of Life Scale (SS-QoL 2.0) developed by Williams et al. [16] is one of the most comprehensive [17] and frequently used patient-reported stroke-specific outcome measure [6, 18, 19]. The language dimension and the patient approach used in the process of item development in the SS-QoL 2.0 scale also make it a scale of choice [6]. It comprises 49 items in 12 domains of self-care, vision, language, mobility, work, upper extremity function, thinking, personality mood, family roles, social roles and energy. The scale is s suitable for mild to moderate stroke of 1 to 6 months’ duration according to previous reports [20–22]. The SS-QoL 2.0 has been cross-culturally adapted to many languages. These include Spanish, German, Danish, Greek, Brazilian, Chinese, Turkish, Kannada (India)**,** Persian, Malayalam (India), Hindi (India) and Yoruba.

Nigeria is a multilingual nation with Hausa, Igbo and Yoruba languages as the three major languages. Hausa is the Chadic language of Afro-asiatic descent with the largest number of speakers. It is spoken as first and second languages by an approximate total of 43 million people (National Population Commission) [23, 24]. Hausa language is spoken across southern Niger and Northern Nigeria and it has developed into lingua franca across most parts of West Africa for trading purposes. There are more than twelve Hausa dialects across the northern part of Nigeria with Kano dialect; Kananci as the standard [24].

The SS-QoL 2.0 has been cross-culturally adapted into Yoruba, the indigenous language of the people of South-western Nigeria using the guidelines of American Association of Orthopaedic Surgeons (AAOS) by Akinpelu et al. [25], and validated by Odetunde et al. [26]. The AAOS guidelines have been used by other authors to conduct cross-cultural adaptation of SS-QoL 2.0 to other languages. These include German [27], Danish [6], Brazilian [28], Spanish [29] and Dutch [30] among other languages. Non-availability of other Nigerian language versions of SS-QoL 2.0 other than the Yoruba version might have limited its utilization in Nigeria. The availability of psychometrically sound translated versions of standardized health measuring scales such as the SS-QoL 2.0 in indigenous Nigerian languages may promote the use of the scales in Nigeria and ensure that Nigerians who are not literate in English are not excluded from measurement of health variables that are important and meaningful to them. This study was therefore aimed at cross-cultural adaptation and validation of SS-QoL 2.0 into Hausa in order to promote its wide availability and utility all over the country and other places where the Hausa language is spoken.

## Method

### Cross-cultural adaptation of SS-QoL 2.0 into Hausa language

Cross-cultural adaptation of SS-QoL 2.0 into Hausa language was carried out following the guidelines of AAOS described by Beaton et al. [10]. The English version of SS-QoL 2.0 was translated to Hausa language by two independent bilingual translators whose mother tongue is Hausa. For the purpose of this study a person’s mother tongue is the indigenous language spoken in his locality of origin. They produced two forward translations (T1 and T2). The two translators are Hausa language lecturers at the School of Languages, Sa’datu Rimi College of Education, Kano. Only one of the translators was provided with information on the purpose and the concept involved in the SS-QoL 2.0 scale. The two Hausa translators met to review the translations T1 and T2 after which a consensus translation T12 was produced. A third bilingual translator (a physiotherapy lecturer at Bayero University, Kano) served as the scribe during the consensus translation. The consensus Hausa translation (T12) of SS-QoL 2.0 was back translated to English by two independent translators who were totally blinded to the process of forward translation. The first back-translator is a physiotherapy clinician who works at Sanni Abacha Specialist Hospital, Damaturu, Yobe state. The second back-translator is a physiotherapy clinician who works at Murtala Muhammed Specialist Hospital, Kano. Two back translations BT1 and BT2 were produced.

An expert committee was set up to review all the translated versions. The members of the expert committee comprised one forward translator (the lecturer at Bayero University, Kano) and one back translator (Physiotherapy clinician at Sanni Abacha Specialist Hospital, Damaturu, Yobe state). Other members of the committee were a physiotherapy lecturer with over 10 years’ experience in health outcomes assessment (University of Ibadan), two physiotherapists currently involved in the treatment of stroke survivors, who were familiar with outcome measurement research, and the researcher who served as the secretary to the committee. Each member of the committee was provided with the original English version of SS-QoL 2.0 scale, translations 1 and 2 (T1 and T2), the consensus translation (T12) and back-translations 1 and 2 (BT1 and BT2). The committee discussed clarity, relevance, and equivalence between the forward and back translations and the original version of the SS-QoL 2.0 by looking at the instructions, response options and the items one after the other. At the end of this first expert committee meeting, the pre-final Hausa version of SS-QoL 2.0 (PFV) was produced.

The PFV was thereafter pretested on thirty Hausa stroke survivors, followed by cognitive debriefing interview by a trained bilingual research assistant to assess relevance, clarity and comprehension of the PFV. Participants’ response to the debriefing interview were considered in a second meeting of the expert committee and necessary modifications were made to the PFV based on feedback from the debriefing interview to produce the final Hausa version of the SS-QoL 2.0 (FHV).

## Results

### Findings from expert committee meetings

The expert committee observed at the first meeting that most of the items in Hausa translation of SS-QoL 2.0 captured the concept of interest as provided in the English version. However, back translation revealed that 12 expressions/items were inconsistent with the original English version and were therefore corrected at this meeting. With all corrections done by the expert committee, PFV was produced. The findings and steps taken by the expert committee to resolve the inconsistencies are summarised in Table 1. The PFV was thereafter taken through pre-testing and cognitive debriefing interview.

**Table 1:** Findings from Expert Panel meetings on Hausa version of SS-QoL 2.0 scale

Original items	Consensus Translation	Back-translation (BT)	Problems	Resolution
1.The title of the scale Stroke-Specific Quality of life scale	Shanyewar barin jiki: tambayoyi a kan kebantattun hanyoyin gudanar da rayuwa. Kashi na 2.0	BT1: Questions about exclusive ways to conduct life, Part2“ BT2: Paralysis of one side of the body: questions concerning activities of daily living”.	BT did not capture the fact that the scale is stroke-specific. This was seen to as reflection of inadequacy in the forward translation	Translation of the title of the scale was modified to capture ‘stroke specific’
2..Response option,- ‘*some trouble’*	Na iyayi da kyar	BT1: “I did it but hardly” BT2:“I can do it but with difficulty”	The BT1 is misleading because *‘hardly’* can also mean *‘rarely’* which does not reflect the concept in the English version. BT2 did not show comparable differences as indicated in the English version.	Hausa translation of the response option was recast to be consistent with the desired concept
3. SC2, “*Do you have trouble eating? For example cutting food or swallowing”*	Ka samu matsala wajen tauna ko hadiyar abinci?	BT1: Did you encounter any problem chewing or swallowing food? BT2: Did you have any problem with chewing or swallowing during eating?	‘for example’ was omitted in the translation while cutting was replaced with chewing	The omitted word was not necessary to convey the intend meaning, while cutting was not applicable to Hausa local food
4. SC4.Did you have trouble getting dressed? For example, putting on socks or shoes, buttoning buttons or zipping.	Ka samu matsala wajen sa kaya? Kamar sa safa ko takalmi ko sa maballi da jan zif na riga?	Did you encounter problem wearing your clothes? For example wearing socks, shoes, and buttons or zipping the dress?	‘Zipping the dress’ was used because the pair of trouser of native wear for Hausa men uses ‘cloth belt’ and not zip	‘Tying up your trousers’ was added to examples of dressing.
V1, “Did you have trouble seeing the television well enough to enjoy a show?”	Ka samu cikas wajen jin dadin kallon talabijin?	Did you have any problem with watching television”	The concept “well enough to enjoy a show” was not captured in the two BT	This was corrected
6.V3; “Did you have trouble seeing things off to one side”	Ka samu matsalar ganin abubuwa da idanunka ba daidai ba?	BT1: “Did you have any difficulty seeing things properly with your eyes” BT2: “Did you have problems with your eyes not seeing some objects very well?”	BT did not capture the concept of ‘seeing from one side’ as contained in the English version	The item was translated again to capture the desired concept.
7.M1 “Did you have trouble walking? (if you can’t walk, circle 1 and go to question M7)”	M1. Ka samu cikas wajen yin tafiya? (idan ba ka iya tafiya to ka kewaye lamba ta M1 sannan ka tafi lamba ta M7)	“Did you have any problem with walking? (If you were not able to walk circle question M1 and move to question M7)	‘question’ M1 instead of response option 1 that was written in the original English version	‘question’ should be removed.
8.UE4, Did you have trouble zipping a zipper’	Ka samu matsalar jan zif/mazagi?	“Did you have problem pulling your zip or rope of the trousers?”	‘Rope’ that is, cloth belt rather than zip is used to tighten trouser by most Hausa men	‘*rope’* should be removed from item UE4 and added to examples of item SC1
9. MD2 ‘I was discouraged about my future’	Na debe haso da rayuwata a gaba	“I lost hope for the future”	‘Discouraged’ and ‘lost hope’ in the BT differ	The item was translated again
10.FR5 ‘I didn’t join in activities just- for- fun with my family’	Ban shiga wasu sha’anoni na farin ciki tare da iyalina ba	BT1:‘I did not have any family activities which interest me’ BT2: ‘I don’t feel happy with my spouse again’	‘*just-for-fun’* was not well translated	The item was translated again.
11.SR1, ‘I didn’t go out as often as I would like’	Ban samu zarafin fita waje sosai kamar yadda nake so ba	BT1: ‘I was not able to be going out as I wanted’ BT2: I didn’t have the opportunity of going out the way I wanted.	BT did not capture the frequency of going out indicated in the original English version	Repeat of translation was done to accommodate this omission.
12.In the section of pre and post stroke ratings, the phrase ‘*before the stroke*’ in the response options	na samu rashin lafiyar	BT1: ‘after I got the ailment’ BT2:‘after the affectation’	BT not consistent with the original English version.	The response option was translated again to be consistent with *‘before the stroke*’ used in the English version

### Findings from pre-testing and cognitive debriefing interview

Participants involved in the cognitive debriefing interview of PFV comprised 20 male and 10 female Hausa stroke survivors. Their mean age was 65.06 ± 9.28 years and mean duration of stroke was 30.03 ± 24.08 months. Participants reported that they understood the items in the PFV. All of them opined that all the items were relevant and no important area was left out. Male participants however stated that item *SC1. ‘Did you have trouble preparing food?’* which was translated as ‘*Kun samu matsala wajen girka abinci?’* was not applicable to them. Their reason was that most Hausa men do not normally get involved in food preparation or do some household chores but they usually go to market to buy foodstuffs. As a result, the expression ‘*ko zuwa sayenkayan abinci’* which means ‘*or going to market to buy foodstuffs’* was added to item SC1 to make it applicable to both the male and the female Hausa stroke survivors. In responding to item SC4, ‘*Did you have trouble getting dressed? For example, putting on socks or shoes, buttoning buttons or zipping* or tying up your trousers’ which was translated as ‘*Ka samu matsala wajen sa kaya? Kamar sa safa ko takalmi ko sa maballi da jan zif na riga/*da mazagi na wando*?* The need to add more examples on female dressing was identified from the comments of female participants. As a result, more examples on female dressing among Hausas such as ‘*tie your wrapper, tie your headscarf or wear your hijab’* which were written as *‘ko daura zane, ko kalabi, ko hijabi’* were added to item SC4 at the second expert committee meeting to produce the FHV. The FHV was thereafter assessed for psychometric properties.

## Psychometric testing of the final Hausa version of the SS-QoL 2.0 scale

### Participants

Eighty-six consenting Hausa stroke survivors (48 males and 38 females) who were receiving physiotherapy in two tertiary health institutions at the Northern part of Nigeria participated in the study. The sample size of at least 50 patients recommended by Altman et al. [31] and Terwee et al. [32] for psychometric assessment of survey instrument was followed.

### Instruments

Instruments used in this study were:

1. Stroke-Specific Quality of Life Scale Version 2.0 developed by Williams et al. [16] Additional file 1.
2. Hausa version SS-QoL 2.0 scale: This was produced at the end of the first stage of the current study.
3. Hausa version of WHOQoL-BREF: This was produced by cross-cultural adaptation of the WHOQoL-BREF [33] into Hausa language by Umar [34]. The scale comprises four domains of physical health (PH), psychological health (PSH), social relationship (SoR) and environment (Env) as well as two items from the overall quality of life and the general health. The Hausa version of WHOQoL-BREF has evidence of construct validity (*r* = 0.51 to 0.64; *p* < 0.001) and test retest reliability (*r* = 0.41 to 0.77; p < 0.001) by [34]. We hypothesised that similar domains of Hausa versions of SS-QoL 2.0 and WHOQoL-BREF would correlate significantly.

### Methods

The research protocol was approved by the University of Ibadan/University College Hospital Health Research Ethics Committee with reference number UI/EC/13/0045. A written informed consent was obtained from each participant. Demographic and clinical data of participants were obtained using a proforma. Convergent validity of the FHV was assessed using the Hausa version of WHOQoL-BREF among 57 Hausa stroke survivors. The FHV and English version of SS-QoL 2.0 scale were administered on 51 of the participants who are literate in English language to assess the construct validity. The FHV was re-administered on 53 of the participants at the interval of 7-days to assess its test-retest reliability. All scales were administered through face to face interview. Face to face interview has been reported to be the least tasking mode of administration of questionnaire which stimulates accurate responses with the interviewer in maximum control of question order [35]. The scales were administered in a random order to eliminate bias.

## Data analysis

Descriptive statistics of mean and standard deviation were used to analyze domains and overall scores on FHV. Convergent, discriminant and construct (comparing scores on FHV and English version of SS-QoL 2.0) validity of the FHV were tested by using the Spearman’s correlation method. Known groups validity by comparing domain scores with gender and age groups was tested using independent t-test and One-way ANOVA respectively. Intra-class correlation (ICC) was used to determine the test-retest reliability and Cronbach’s alpha values were determined for internal consistency of the FHV. SPSS version 16.0 (Chicago IL SPSS Inc.) was used to analyze data. Level of significance was set at *p* < 0.05.

## Results

### Demographic and clinical characteristics of Hausa participants

The mean age of participants was 60.00 ± 12.13 years and the mean duration since stroke was 20.0 ± 20.4 months (median duration of 12 months). The socio-demographic and clinical characteristics of the respondents are presented in Table 2. The mean age of participants at onset of stroke was 58.33 ± 11.54 years. The majority (86%) of stroke survivors in this study belonged to age group 50 years and above at the onset of stroke. The distribution of participants by hemiplegic/ hemiparetic sides was about equal for the right and left sides. Fifty-one percent of the participants had right hemiplegia/hemiparesis. Most participants (85%) have had stroke for more than 6 months.

**Table 2:** Demographic and Clinical Characteristics of Hausa Stroke Survivors

Variables	Frequency	Percentage
Sex		
Male	47	55
Female	39	45
Age group (years)		
< 50	12	14
50–59	26	30
60–69	29	34
> 70	19	22
Age at onset of stroke		
< 50	1214	
50–59	25	29
60–69	30	35
> 70	19	22
Affected side		
Left	42	49
Right	44	51
Time since Stroke onset		
< 3 months	4	5
3–6 months	9	10
> 6 months	73	85

### Construct validity of Hausa version of SS-QoL 2.0

Comparison of Participants’ scores on English and Hausa Versions of SS-QoL 2.0 showed that participants’ mean overall score on the English version of SS-QoL 2.0 (145.30 ± 39.78) did not differ significantly from the mean overall score on the FHV (150.41 ± 40.45). The domains score of the English of SS-QoL 2.0 and FHV did not differ significantly except in self-care and work domains (Table 3). There were good to excellent and significant correlations in participants’ mean overall and each of the domains’ scores between the English version of SS-QoL 2.0 and FHV (Table 4).

**Table 3:** Wilcoxon’s Signed Rank Test of Participants’ Scores on the English and Hausa versions of SS-QoL 2.0 scale

Domains		Mean	Standard Deviation	*z* value	*p* value
Self-care (SC)	English	14.3	5.08	−2.04	0.04*
	Hausa	14.81	5.34		
Vision (V)	English	9.41	3.09	−0.78	0.44
	Hausa	9.57	3.18		
Language (L)	English	15.89	4.89	−0.36	0.72
	Hausa	16.14	4.93		
Mobility (M)	English	15.62	7.96	−1.75	0.08
	Hausa	16.32	8.25		
Work (W)	English	8.46	2.92	−2.35	0.02*
	Hausa	9.03	3.30		
Upper extremity (UE)	English	14.05	5.38	− 1.5	0.14
	Hausa	14.76	5.67		
Thinking (T)	English	8.86	2.82	−1.03	0.3
	Hausa	9.05	2.68		
Personality (P)	English	9.16	2.86	−1.61	1.11
	Hausa	9.43	2.83		
Mood (MD)	English	15.92	4.68	−1.1	0.27
	Hausa	16.24	4.42		
Family role (FR)	English	9.32	2.61	−1.78	0.08
	Hausa	9.81	3.01		
Social Role (SR)	English	14.86	4.47	−0.67	0.51
	Hausa	5.08	4.19		
Energy (E)	English	9.43	2.90	−1.2	0.23
	Hausa	9.89	2.96		
Overall (O)	English	145.30	39.78	−1.08	0.28
	Hausa	150.41	40.45

**Table 4:** Spearman’s Correlation of Participants’ Scores on English and Hausa versions of SS-QoL 2.0 scale

Domains	r-value	*p*-value
Self-Care	0.92	0.001^*^
Vision	0.87	0.001^*^
Language	0.87	0.001 ^*^
Mobility	0.87	0.001^*^
Work	0.88	0.001^*^
Upper Extremity	0.86	0.001^*^
Thinking	0.84	0.001^*^
Personality	0.83	0.001^*^
Mood	0.77	0.001^*^
Family role	0.70	0.001^*^
Social role	0.78	0.001^*^
Energy	0.73	0.001^*^
Overall	0.88	0.001^*^

^*^significant correlation *p* < 0.05

For the known-group validity of the FHV by gender and age, Table 5 shows the result of the independent t-test comparison of domains and overall scores by gender. The result showed no significant gender difference in the domains and overall scores (*p* > 0.05), although the study may be underpowered to detect a difference in domain scores based on gender. Table 6 shows the result of the One-way ANOVA comparison of domains and overall scores by age group. There were no significant differences in the mean domains and overall scores on FHV for 7 out of the 12 domains across various age groups (p > 0.05); while 5 domains (self-care, vision, language, mobility, upper extremity role and the overall score) demonstrated significant differences across various age groups (*p* < 0.05). Highest mean scores on all domains (except personality domain) and the overall score were observed for the lowest age group (< 50 years). Whereas, lowest mean scores were observed for the oldest age group (> 70 years) on all domains (except thinking and energy domains) and the overall score. The details for item-domain correlations (discriminant validity) for FHV are presented in Table 7. The result showed that item-domain correlations (i.e., correlations of an item with its own domain) were comparable within each domain of FHV. There were significant correlations between all the domains of FHV as well as between the overall score and each of the domains’ score. Items in all the twelve domains had correlation scores > 0.8 (r ranged from 0.803–0.979) with their own domains. Most items had correlation scores greater than 0.20 with other than their own domains except a few items (details in Table 7).

**Table 5:** Independent t-test comparison of domains and overall score of the Final Hausa version of the Stroke-specific Quality of Life Scale by gender

Domains	Gender		*t*-value	*p*-value
	Male x̄ ±SD(*n* = 47)	Female x̄ ±SD(*n* = 39)		
SC	14.98 ± 5.06	15.54 ± 5.41	−0.50	0.62
V	10.45 ± 3.53	10.18 ± 3.49	0.35	0.73
L	18.64 ± 5.83	18.36 ± 5.94	0.22	0.83
M	17.85 ± 8.17	16.79 ± 7.80	0.61	0.54
W	8.55 ± 3.43	8.46 ± 3.34	0.13	0.90
UE	13.28 ± 5.90	14.05 ± 5.92	0.61	0.55
T	8.89 ± 3.79	8.46 ± 3.89	0.52	0.60
P	8.11 ± 3.68	8.62 ± 3.89	−0.62	0.54
MD	15.63 ± 5.44	15.26 ± 5.29	0.33	0.74
FR	8.98 ± 3.74	9.46 ± 3.51	−0.61	0.54
SR	12.30 ± 6.00	14.28 ± 5.71	−1.56	0.12
E	8.51 ± 3.78	8.69 ± 3.70	− 0.22	0.82
O	146.17 ± 41.64	148.41 ± 40.66	− 0.25	0.80

Alpha level was set at *p* < 0.05

*SC*-Self Care, *V*-Vision, *L*-Language, *M*-Mobility, *W*-Work, *UE*-Upper Extremity, *T*-Thinking, *P*-Personality, *MD*-Mood, *FR*-Family Role, *SR*-Social Role, *E*-Energy, *O*-Overall

**Table 6:** One-way ANOVA comparison of the Hausa version of the Stroke-Specific Quality of Life 2.0 domains by age group

SS-QoL Domains	Age groups					
	< 50 x̄ ±SD (*n* = 16)	50–59 x̄ ±SD (*n* = 22)	60–69 x̄ ±SD (*n* = 29)	> 70 x̄ ±SD (*n* = 19)	*F*-ratio	*p*-value
SC	17.31 ± 4.03	16.00 ± 4.89	15.48 ± 5.10	12.21 ± 5.59	3.454	0.020^*^
V	11.81 ± 2.23	11.09 ± 3.39	10.79 ± 2.96	7.47 ± 3.85	6.906	0.001^*^
L	21.00 ± 4.24	18.95 ± 5.09	19.14 ± 5.79	14.95 ± 6.65	3.818	0.013^*^
M	22.06 ± 5.45	16.86 ± 8.16	18.59 ± 6.33	12.16 ± 9.18	5.581	0.002^*^
W	10.74 ± 3.12	8.68 ± 3.30	8.41 ± 3.42	7.21 ± 3.28	2.093	0.107
UE	16.75 ± 5.76	13.36 ± 5.31	13.97 ± 5.89	10.79 ± 5.64	3.257	0.026^*^
T	9.69 ± 3.57	7.64 ± 3.55	8.90 ± 3.80	8.78 ± 4.33	0.953	0.419
P	8.81 ± 3.97	7.91 ± 3.69	8.86 ± 4.07	7.63 ± 3.27	0.582	0.628
MD	17.06 ± 5.08	15.05 ± 5.16	15.69 ± 5.40	14.26 ± 5.72	0.856	0.468
FR	9.50 ± 3.60	9.23 ± 4.01	9.24 ± 3.42	8.84 ± 3.75	0.097	0.962
SR	15.25 ± 5.42	12.23 ± 6.63	13.45 ± 6.16	12.21 ± 4.98	1.032	0.383
E	9.75 ± 3.47	8.14 ± 3.31	8.38 ± 4.15	8.47 ± 3.76	0.656	0.581
O	169.00 ± 33.79	145.59 ± 42.30	150.90 ± 37.65	125.00 ± 41.42	3.801	0.013^*^

^*^Significant differences. Alpha level was set at *p* < 0.05

*SC*-Self Care, *V*-Vision, *L*-Language, *M*-Mobility, *W*-Work, *UE*-Upper Extremity, *T*-Thinking, *P*-Personality, *MD*-Mood, *FR*-Family Role, *SR*-Social Role, *E*-Energy, *O*-Overall

**Table 7:** Item-domain correlations (discriminant validity) of the Hausa version of the Stroke Specific Quality of Life 2.0 (*n* = 86)

SS-QoL Domain	SC	V	L	M	W	UE	T	P	MD	FR	SR	E
SC1	0.952	0.486	0.383	0.623	0.627	0.651	0.172	0.107	0.340	0.360	0.362	0.362
SC2	0.941	0.507	0.337	0.365	0.647	0.689	0.121	0.118	0.302	0.312	0.332	0.332
SC4	0.888	0.513	0.303	0.617	0.594	0.604	0.186	0.062	0.243	0.286	0.249	0.249
SC5	0.932	0.493	0.326	0.552	0.580	0.650	0.139	0.038	0.315	0.364	0.321	0.321
SC8	0.955	0.493	0.383	0.618	0.608	0.639	0.168	0.075	0.308	0.278	0.271	0.271
V1	0.531	0.922	0.578	0.618	0.433	0.367	0.274	0.143	0.373	0.362	0.384	0.384
V2	0.475	0.916	0.536	0.547	0.391	0.298	0.172	0.177	0.296	0.329	0.327	0.327
V3	0.432	0.955	0.650	0.586	0.346	0.341	0.196	0.167	0.309	0.334	0.112	0.368
L2	0.341	0.630	0.958	0.499	0.298	0.242	0.265	0.220	0.266	0.300	0.128	0.267
L3	0.374	0.599	0.956	0.492	0.321	0.255	0.218	0.142	0.223	0.260	0.108	0.250
L5	0.381	0.684	0.943	0.537	0.354	0.301	0.212	0.265	0.273	0.336	0.113	0.285
L6	0.344	0.625	0.935	0.527	0.324	0.221	0.255	0.265	0.315	0.348	0.137	0.322
L7	0.406	0.534	0.910	0.472	0.309	0.313	0.247	0.213	0.234	0.254	0.154	0.275
M1	0.618	0.631	0.489	0.953	0.803	0.584	0.263	0.326	0.497	0.438	0.398	0.523
M4	0.626	0.612	0.471	0.958	0.790	0.578	0.237	0.288	0.467	0.435	0.397	0.509
M6	0.612	0.595	0.450	0.803	0.776	0.554	0.240	0.271	0.449	0.423	0.393	0.514
M7	0.624	0.572	0.483	0.969	0.786	0.582	0.247	0.283	0.464	0.428	0.405	0.558
M8	0.599	0.606	0.515	0.966	0.785	0.566	0.215	0.270	0.432	0.422	0.412	0.529
M9	0.572	0.623	0.536	0.960	0.761	0.534	0.225	0.229	0.436	0.375	0.373	0.507
W1	0.617	0.351	0.251	0.761	0.964	0.610	0.160	0.269	0.338	0.310	0.482	0.513
W2	0.635	0.386	0.272	0.778	0.958	0.592	0.225	0.313	0.379	0.316	0.483	0.512
W3	0.661	0.397	0.308	0.790	0.957	0.618	0.174	0.284	0.362	0.384	0.469	0.500
UE1	0.676	0.373	0.272	0.600	0.641	0.957	0.129	0.296	0.394	0.453	0.431	0.416
UE2	0.712	0.365	0.287	0.606	0.666	0.969	0.157	0.270	0.370	0.439	0.446	0.400
UE3	0.685	0.343	0.279	0.578	0.667	0.973	0.198	0.320	0.396	0.456	0.456	0.423
UE4	0.670	0.332	0.248	0.558	0.638	0.964	0.207	0.301	0.412	0.469	0.403	0.425
UE5	0.642	0.311	0.240	0.465	0.522	0.904	0.150	0.318	0.354	0.364	0.317	0.328
T2	0.183	0.189	0.206	0.325	0.125	0.256	0.964	0.485	0.636	0.486	0.581	0.567
T3	0.141	0.196	0.237	0.246	0.095	0.178	0.979	0.436	0.617	0.444	0.512	0.519
T4	0.165	0.266	0.272	0.262	0.077	0.180	0.963	0.440	0.581	0.445	0.453	0.512
P1	0.082	0.127	0.163	0.298	0.308	0.377	0.415	0.973	0.615	0.642	0.539	0.576
P2	0.040	0.150	0.208	0.302	0.297	0.306	0.450	0.972	0.589	0.616	0.521	0.567
P3	0.080	0.214	0.230	0.277	0.247	0.247	0.436	0.969	0.625	0.618	0.513	0.586
MD2	0.344	0.325	0.272	0.469	0.362	0.430	0.603	0.606	0.960	0.738	0.589	0.715
MD3	0.329	0.350	0.264	0.483	0.429	0.424	0.642	0.598	0.970	0.771	0.609	0.739
MD6	0.303	0.339	0.239	0.445	0.405	0.412	0.646	0.573	0.953	0.779	0.627	0.752
MD7	0.310	0.391	0.252	0.479	0.343	0.409	0.637	0.612	0.955	0.770	0.564	0.710
MD8	0.295	0.359	0.286	0.461	0.395	0.375	0.625	0.595	0.947	0.730	0.509	0.657
FR5	0.339	0.361	0.304	0.409	0.383	0.463	0.466	0.581	0.720	0.961	0.620	0.603
FR7	0.364	0.302	0.246	0.423	0.393	0.515	0.445	0.617	0.760	0.955	0.594	0.619
FR8	0.281	0.297	0.234	0.418	0.334	0.441	0.408	0.639	0.745	0.961	0.594	0.632
SR1	0.340	0.065	0.046	0.379	0.474	0.481	0.465	0.473	0.577	0.633	0.955	0.649
SR4	0.321	0.094	0.096	0.412	0.499	0.446	0.498	0.514	0.567	0.617	0.962	0.662
SR5	0.291	0.119	0.074	0.405	0.492	0.454	0.556	0.544	0.600	0.616	0.964	0.687
SR6	0.341	0.093	0.132	0.417	0.523	0.492	0.506	0.621	0.602	0.629	0.959	0.668
SR7	0.356	0.122	0.140	0.410	0.527	0.478	0.529	0.565	0.556	0.603	0.956	0.676
E1	0.283	0.356	0.233	0.514	0.535	0.397	0.484	0.622	0.645	0.606	0.458	0.967
E2	0.326	0.410	0.288	0.538	0.543	0.436	0.534	0.568	0.732	0.649	0.537	0.974
E3	0.362	0.392	0.285	0.568	0.543	0.495	0.567	0.563	0.755	0.666	0.326	0.958

*SC*-Self Care, *V*-Vision, *L*-Language, *M*-Mobility, *W*-Work, *UE*-Upper Extremity, *T*-Thinking, *P*-Personality, *MD*-Mood, *FR*-Family Role, *SR*-Social Role, *E*-Energy

### Correlation of participants’ scores on Hausa versions of SS-QoL 2.0 scale and WHOQoL-BREF (convergent validity)

Domains with similar items on the Hausa versions of SS-QoL 2.0 scale and WHOQoL-BREF were compared. The correlation between Physical Health (PH) domain on Hausa version of WHOQoL-BREF and mobility, work and energy domains of FHV were moderate to good (*r* = 0.46, 0.61 and 0.50 respectively) at *p* < 0.05. The correlation between Psychological Health (PSH) domain on Hausa version of WHOQoL-BREF and thinking and mood domain of FHV were weak to moderate (*r* = 0.21 and 0.36 respectively) at p < 0.05 for mood domain. Social Relationship (SoR) domain of Hausa version of WHOQoL-BREF correlated with Social Role domain of FHV with low r value of 0.09 while Environment domain on Hausa version of WHOQoL-BREF showed moderate r values of 0.36 and negligible r value of 0.05 with family role and social role domains on FHV respectively at p < 0.05 for family role domain (Table 8). The overall scores on FHV showed moderate to good (*r* = 0.31 to 0.62) and significant correlation with the Physical Health, Psychological Health and Environment domains of Hausa version of WHOQoL-BREF and weak (*r* = 0.14) correlation with the Social Relationship domain. Correlations were interpreted as negligible (< 0.1) small or weak (0.1 to < 0.3), medium or moderate (0.3 to < 0.5) and large or good (≥ 0.5) [36]

**Table 8:** Spearman’s Correlation of Hausa versions of SS-QoL 2.0 and WHOQoL-BREF

Domains	WHOQoL-BREF							
	PH		PSH		SoR		Env	
SS-QoL 2.0	*r*	*p*	*r*	*p*	*r*	*p*	*r*	*p*
M	0.46	0.001*	–	–	–	–	–	–
W	0.61	0.001*	–	–	–	–	–	–
T	–	–	0.21	0.11	–	–	–	–
MD	–	–	0.36	0.006*	–	–	–	–
FR	–	–	–	–	–	–	0.36	0.006*
SR	–	–	–	–	0.09	0.49	0.05	0.70
E	0.50	0.001*	–	–	–	–	–	–
O	0.62	0.001*	0.43	0.001*	0.14	0.31	0.33	0.01*

* indicates significant correlation (*p* < 0.05)

- indicates dissimilar domains on the two scales

*PH*- Physical Health; *PSH*-Psychological Health; *SoR*-Social Relationship; *Env*-Environment; *M*-mobility; *W*-work; *T*-thinking; *MD*-mood; *FR*-family role; *SR*-social role; *E*-energy; *O*-overall

### Reliability of Hausa version of SS-QoL 2.0 scale

Cronbach’s alpha value for the domains of FHV ranged from 0.71 to 0.90 (Table 9). Intra-class Correlation Coefficient was 0.98 for the overall score while the domains’ score ranged from 0.86 to 0.99 at *p* value less than 0.01 (Table 10). Other psychometric properties tested were the floor and ceiling effects of the FHV which indicated that none of the domains and the overall score demonstrated significant (less than 20%) floor or ceiling effects (Table 11). Cronbach’s alpha values above 0.9 is excellent, above 0.8 is good, above 0.7 is acceptable, above 0.6 is questionable, above 0.5 is poor, and below 0.5 is unacceptable. [37]

**Table 9:** Cronbach’s alpha values of Hausa version of SS-QoL 2.0 scale

Domains	Number of items	Mean ± SD	Cronbach’s alpha
Self Care	5	15.23 ± 5.20	0.82
Vision	3	10.33 ± 3.49	0.77
Language	5	18.51 ± 5.85	0.76
Mobility	6	17.37 ± 7.98	0.90
Work	3	8.51 ± 3.37	0.86
Upper Extremity	5	13.63 ± 5.89	0.85
Thinking	3	8.70 ± 3.82	0.71
Personality	3	8.34 ± 3.76	0.74
Mood	5	15.47 ± 5.34	0.87
Family Role	3	9.20 ± 3.62	0.85
Social Role	5	13.20 ± 5.92	0.82
Energy	3	8.59 ± 3.72	0.87

*SD*-Standard Deviation

**Table 10:** Intra-class Correlation between scores on Hausa version of SS-QoL 2.0 scale on two occasions

Domains	ICC	95% CI	
		Lower bound	Upper bound
Self Care	0.92	0.86	0.95
Vision	0.94	0.90	0.96
Language	0.96	0.94	0.98
Mobility	0.97	0.95	0.98
Work	0.95	0.92	0.97
Upper Extremity	0.95	0.92	0.97
Thinking	0.99	0.98	0.99
Personality	0.87	0.78	0.92
Mood	0.86	0.77	0.92
Family Role	0.86	0.77	0.92
Social Role	0.98	0.97	0.99
Energy	0.98	0.97	0.99
Overall	0.99	0.96	0.99

*ICC*-Intra-class Correlation

*CI*-Confidence Interval

**Table 11:** Percentage of participants with Minimum and Maximum scores on Hausa Version of SS-QoL 2.0 scale (Floor/ Ceiling Effects)

Domains	Effects	
	Floor (%)	Ceiling (%)
Self-care	5.8	2.3
Vision	4.7	8.1
Language	2.3	19.8
Mobility	4.7	5.8
Work	11.6	3.5
Upper extremity	1.2	5.8
Thinking	1.2	1.2
Personality	1.2	5.8
Mood	3.5	4.7
Family role	9.3	7.0
Social role	1.2	1.2
Energy	9.3	4.7
Overall	1.2	1.2

## Discussion

Hausa is one of the three major and most common Nigerian languages spoken by 37%, of the population [23]. Hausa is also spoken by about 43 million people as a first and second language. The language is spoken in Niger, Nigeria, Benin, Ghana, Cameroon and Sudan [24]. The process of cross-cultural adaptation of SS-QoL 2.0 into Hausa language in this study was according to the guidelines recommended by American Association of Orthopaedic Surgeons (AAOS) [10]. In the forward translation of the SS-QoL 2.0 to Hausa language, some words such as ‘zip’ and ‘button’ which have no equivalent in Hausa language had to be used as ‘loan’ or ‘borrowed’ words. Zip was written as ‘zif’ in Hausa language. This was also the case in cross-cultural adaptation of SS-QoL 2.0 into Yoruba language in which the words ‘zip’ and ‘button’ were written as ‘siipu’ and ‘bọtini’ [26]. Back-translation of the Hausa version of SS-QoL 2.0 scale revealed discrepancies, inconsistencies and omission in twelve items/expressions. These discrepancies were resolved by the expert committee (details in Table 1).

This study assessed the construct, convergent, discriminant and known group validity, test-retest reliability and internal consistency of the FHV. The mean age of stroke survivors who participated in this study was 60.00 ± 12.13 years with median time since stroke of 12 months. The mean age of participants at onset of stroke was 58.33 ± 11.54 years. These findings support the earlier reports that stroke occurs more frequently in middle age [21, 22, 26, 28, 38–41]. Strokes can occur at any age, though its risk increases with age, [42] while stroke duration varies extensively among the survivors. This may be attributed to factors such as presence of co-morbidities, quality of care and access to care and rehabilitation, racial and ethnic disparities, perceived social support and psychological state among other factors [43]. Sex distribution of participants in this study also indicated a male preponderance, consistent with findings from previous studies [21, 22, 25, 27],[38, 39, 43]. The distribution of participants by hemiparetic sides was about equal for the right and left sides. Fifty-one percent of the participants had right hemiplegia/hemiparesis while 49% had left hemiplegia/hemiparesis. Left hemispheric ischaemic stroke presenting with right hemiplegia has been reported to be more frequent than right hemispheric cases [44]. The close proportion reported for right and left hemiplegia/hemiparesis reported in this study may be due to the sample size and the use of only two hospitals for data collection. A similar study by Odetunde et al. [26] involved 100 stroke survivors across 8 different hospitals.

Hausa version of the SS-QoL 2.0 demonstrated good to excellent test-retest reliability and high internal consistency or homogeneity of the domains in agreement with homogeneity of the domains of SS-QoL 2.0 scale reported by Hsueh et al. [45]. The findings from this study are further supported by findings from previous studies on different versions of SS-QoL 2.0 scale. Williams et al. [16] and Boosman et al. [21] reported excellent internal consistency for ischaemic stroke survivors and patients with aneurysmal subarachnoid haemorrhage respectively. Similarly, Kerber et al. [46] reported excellent test retest reliability and internal consistency for SS-QoL 2.0 scale in English speaking Mexican American population. The Danish version was reported to have moderate to excellent test retest reliability and excellent internal consistency by Muus et al. [20]. The Brazilian version of SS-QoL 2.0 was reported by Lima et al. [28] to have excellent test-retest reliability while the Spanish version of SS-QoL 2.0 showed good to excellent test retest reliability and internal consistency [47, 48]. Harkverdioglu and Khorshid [49] reported excellent test-retest reliability and internal consistency for Turkish version of SS-QoL 2.0, while the Chinese version of SS-QoL 2.0 demonstrated good to excellent internal consistency according to Wong et al. [50].

The Hausa version of the SS-QoL 2.0 demonstrated evidence of same construct as the English version. This is further supported by the finding that participants’ overall and almost all (ten out of twelve) domains scores on the English and Hausa versions of the SS-QoL 2.0 did not differ significantly. Differences or lack thereof could however be interpreted several ways, and imply that the adaptation was successful or not successful entirely based on how differences are interpreted (e.g. very similar scores therefore an excellent translation; or conversely, a failure to adequately capture differences). Coincidentally, mean domain scores were highest on mood domain and lowest on work domain on both versions of SS-QoL 2.0 scale. Our findings may imply that the FHV is a valid translation of the English version of the SS-QoL 2.0 and provides an evidence of construct validity of FHV. Similar findings were reported by Akinpelu et al. [25] and Odetunde et al. [26] for Yoruba version of SS-QoL 2.0 scale.

The result of test of known-group validity of the FHV indicated that there was no significant gender difference between the domains and overall scores. This is consistent with report of Xie et al. [43] and Zalihic et al. [51]. Male participants had higher mean score in vision, language, mobility, work, thinking and mood domains while female participants had higher mean score in self-care, upper extremity, personality, family role, social role and energy domains and the overall QoL score. Conversely, previous studies reported significantly lower post-stroke QoL in females and that females were more negatively affected in their QoL [1, 52, 53]. In these studies, QoL was assessed in different settings from the current study using different QoL scales, mode of administration and stroke patients, and these factors may be responsible for the observed differences in QoL and gender. Nevertheless, the finding of this study should be treated with caution as it might be underpowered to detect a difference in domain scores based on gender. Given the difference scores between males and females on some domains like SR which may be interpreted as inconclusive.

Our study also showed that the domains of FHV were associated with age. The mean domain scores differ significantly across the four age groups on self-care, vision, language, mobility, upper extremity and overall score. Significant influence of age on QoL scores in the self-care, mobility and upper extremity functions domains is not surprising, as these domains are the underlying component of the physical domain sub-scales of SS-QoL2.0. Left hemispheric stroke which manifests as right hemiparesis/hemiplegia would most likely cause communication difficulties and may explain the reason for significant influence of QoL scores on Language domain of FHV. This is similar to the report of Nichols-Larsen et al. [54]. Significant difference across age groups and vision domain may be explained in terms of visual impairments that are sometimes associated with stroke, effect of which might not have been overcome by the stroke-survivors. In a like manner, post-stroke difficulties in visual function has been reported to cause significant impact to the quality of life of stroke survivors [55]. The reported differences in the QoL scores among participants is not significant on the remaining 7 domains of FHV but has demonstrated that age of stroke survivors was an important factor that determines their HRQoL [54, 56–58].

All items in the FHV demonstrated strong correlations with its own domain than with domains measuring other concepts. For example, item M7 ‘Did you have trouble with needing to stop and rest when walking or using a wheelchair?’ had r value of 0.969 with its own domain, while the same item had r value of 0.247 with thinking domain. This finding of strong correlations of items with its own domain is consistent with reports of Muus et al. [20] and Cruz-Cruz et al. [43] which describes the relevance of the items in their respective domains and the discriminant validity of the FHV.

The WHOQoL-BREF was the only Hausa quality of life scale available to the researcher as at the time of data collection for this study. The finding of moderate to good correlation of FHV with all domains of Hausa version of WHOQoL-BREF indicates that the overall score of the FHV represents the overall status of HRQoL and is consistent with the report of Hsueh et al. [46] and Williams et al. [16]. This link between overall SS-QoL score and the overall status of HRQoL of stroke survivors is useful in capturing the multiple impacts of stroke from the patients’ point of view. Correlation between similar domains on the two scales can be explained in terms of correlation between related domains where in most cases domains of SS-QoL 2.0 are components of domains of WHOQoL-BREF. Boosman et al. [21] reported similar findings of significant correlation between the Cognitive Failures Questionnaire (CFQ), Life Satisfaction-9 (LiSat-9) and Hospital Anxiety Depression Scale (HADS) and corresponding SS-QoL 2.0 domain scores. The SS-QoL 2.0 scale has been compared with various other measures with varying findings. Muus et al. [20] reported moderate to excellent correlation (r ranging from 0.37 to 0.88) between the domains of Danish version of SS-QoL 2.0 and the Barthel Index and National Institute of Health Stroke Scale. Similar to the findings of the present study, Boosman et al. [21] reported that SS-QoL 2.0 scores showed weak to moderate correlations (0.24–0.32) with the Glasgow Outcome Scale and moderate to strong correlations (0.35–0.72) between SS-QoL 2.0 scores and CFQ, LiSat-9 and HADS. Lin et al. [22] also reported weak to moderate correlations (r ranging from − 0.04 to 0.52) between the domains of the SS-QoL 2.0 and Fugl-Meyer Assessment, Functional Independent Measure, and Frenchay Activities Index with **excellent** correlation (*r* = 0.65) only between the SS-QoL 2.0 Self-Care domain and Functional Independent Measure. The findings in the present study are consistent with findings in the reported studies. The fact that the correlations between similar domains of Hausa versions of SS-QoL 2.0 and WHOQoL-BREF are statistically significant may be evidence that the Hausa version of SS-QoL 2.0 scale capture relevant QoL items which are also assessed by the WHOQoL-BREF. Low correlation coefficient between some similar domains on the WHOQoL-BREF and SS-QoL 2.0 may be due to certain factors. Previous studies that compared the SS-QoL with generic scales used more than one scale for comparison such that most related domains on the SS-QoL and the other scales were compared [20, 27, 48, 49, 59]. In the current study however, the domains of WHOQoL-BREF only were used to compare the domains of SS-QoL 2.0 and this may not allow for comparison with most related domains as obtained in previous studies. The process of social adaptation has been reported to be a possible factor that can cancel out supposed differences in QoL. This fact can also explain in like manner the apparent weak correlation between the generic measure, WHOQoL-BREF and a specific measure SS-QoL 2.0 [20, 49]. The items in each domain of SS-QoL 2.0 were structured in a specific and simplified manner that addresses stroke-related problems, but this is not so for the generic WHOQoL-BREF. The differences in the contents of the two scales may also explain the weak correlations between these compared domains. The FHV 2.0 demonstrated no significant floor or ceiling effects on any of its domains or the overall scores. Absence of floor effect implies that FHV can discriminate between varying degrees of stroke severity and can identify stroke survivors with severe stroke. Absence of ceiling effect implies that the scale can also identify any level of improvements in condition of stroke survivors in any of the QoL domains. This is in line with findings of Williams et al. [16] and Cruz-Cruz et al. [43].

This study has some limitations that may need to be considered in interpreting and generalizing its findings. First, with regards to the revision made to item SC1, preparing food and going to the market to buy foodstuffs are not equivalent. They are different activities requiring different amounts of effort or exertion. This revision was however done to make the item applicable to both male and female participants. Future study should consider what else could be done to address this very interesting cultural issue/gender difference. Second, participants were 20.0 ± 20.4 month post-stroke. This suggests that the results may have limited generalizability to patients early in the stroke recovery, patients at different phases of post-stroke recovery may have different HRQoL issues and it is not clear that the FHV has demonstrated that validity. Future research is recommended to address this limitation. Third, participants in this study received different rehabilitation programs throughout the duration of the study; further research is needed to assess the psychometric properties of the FHV for specific treatment programs on larger samples to provide further insights into the psychometric properties of the FHV in particular situations. Fourth, there are possible differences in psychometric properties in patient-reported QoL outcomes due to the modes of administration [60], thus further research may be needed to study psychometric properties of the FHV using different modes of administration such as paper-and-pencil administration at home, via the mail and telephone interview. Lastly, in this study, the FHV was validated using the Hausa version of WHOQoL-BREF, other Hausa outcome measures with the advantage of assessing domains relevant to stroke, should be used to compare the domains of FHV in order to further ascertain its validity with other established outcome measures.

## Conclusion

The English version of SS-QoL 2.0 was successfully cross-culturally adapted to Hausa language. The FHV captured the concept of interest as the English version. Test-retest reliability, internal consistency, discriminant, construct, convergent and known-group validity of the FHV are adequate. Hausa version of the SS-QoL 2.0 is therefore a valid and reliable measure with no floor or ceiling effects and recommended for assessing health-related quality of life among Hausa stroke survivors.

## Additional files

Additional file 1: Final Hausa version of the SSQoL 2.0 (DOCX 39 kb)

## Abbreviations

BT1: Back-translation 1
BT2: Back-translation 1
CFQ: Cognitive Failure Questionnaire
CI: Confidence Interval
E: Energy
Env: Environment
FHV: Final Hausa version of SS-QoL
FR: Family Role
HADS: Hospital Anxiety and Depression Scale
HRQoL: Health Related Quality of Life
ICC: Intra- class Correlation Coefficient
L: Language
LiSAT: Life Satisfaction Questionnaire
M: Mobility
MD: Mood
O: Overall
P: Personality
PFV: Pre-final Hausa version of SS-QoL
PH: Physical Health
PSH: Psychological Health
QoL: Quality of Life
SC: Self Care
SD: Standard deviation
SoR: Social Relationship
SPSS: Statistical Package for Social Sciences
SR: Social Role
SS-QoL 2.0: Stroke-Specific Quality of Life scale version 2.0
T: Thinking
T1: Translation 1
T12: Consensus Translation
T2: Translation 2
UE: Upper Extremity
V: Vision
W: Work
WHOQoL-BREF: World Health Organisation Quality of Life short form
