# Supplementary material for: Cross-cultural adaptatiion and validation of the stroke specific quality of life 2.0 scale into Hausa language
Source: J Patient Rep Outcomes. 2018 Dec 20;2:63. doi: 10.1186/s41687-018-0082-1 (PMC6301903; doi:10.1186/s41687-018-0082-1)
Supplement: Supplementary file 1 — Final Hausa version of the SSQoL 2.0 (DOCX 39 kb) [file 41687_2018_82_MOESM1_ESM.docx]

**Stroke-Specific Quality of Life Scale Version 2.0-Original English Version (Williams et al, 1999)**

Instructions:

We will like to know how you are doing with activities or feelings that can sometimes be affected by stroke. Each question will ask about a specific activity or feeling. For each question think about how that activity or that feeling has been for you in the last one week.

The first group of questions asks about how much trouble you have with specific activities. Each question deals with problems that some people hsave after their stroke. Circle a number in the box that best describes how much trouble you have with the activity in the past week.

# DURING THE LAST WEEK:

|  | Couldn’t do it at all  1 | A lot of trouble  2 | Some trouble  3 | A little trouble  4 | No trouble at all  5 |
| --- | --- | --- | --- | --- | --- |
| SC1.Did you have trouble preparing food? | 1 | 2 | 3 | 4 | 5 |
| SC2.Did you have trouble eating? For example cutting food or swallowing? | 1 | 2 | 3 | 4 | 5 |
| SC4.Did you have trouble getting dressed? For example, putting on socks or shoes, buttoning buttons or zipping. | 1 | 2 | 3 | 4 | 5 |
| SC5.Did you have trouble taking a bath or a shower? | 1 | 2 | 3 | 4 | 5 |
| SC8.Did you have trouble using the toilet? | 1 | 2 | 3 | 4 | 5 |
| V1.Did you have trouble seeing the television well enough to enjoy a show? | 1 | 2 | 3 | 4 | 5 |
| V2.Did you have trouble reaching things because of poor eye sight? | 1 | 2 | 3 | 4 | 5 |
| V3.Did you have trouble seeing things off to side? | 1 | 2 | 3 | 4 | 5 |
| L2.Did you have trouble speaking? For example get stuck, stutter, stammer or slur your words? | 1 | 2 | 3 | 4 | 5 |

**DURING THE LAST WEEK**:

|  | Couldn’t do it at all  1 | A lot of trouble  2 | Some trouble  3 | A little trouble  4 | No trouble  at all  5 |
| --- | --- | --- | --- | --- | --- |
| L3.Did you have trouble speaking clearly enough to use the telephone? | 1 | 2 | 3 | 4 | 5 |
| L5.Did other people have trouble in understanding what you said? | 1 | 2 | 3 | 4 | 5 |
| L6.Did you have trouble finding the word you wanted to say? | 1 | 2 | 3 | 4 | 5 |
| L7.Did you have to repeat yourself so others could understand you? | 1 | 2 | 3 | 4 | 5 |
| M1.Did you have trouble walking? (if you can’t walk, circle 1 and go to question M7) | 1 | 2 | 3 | 4 | 5 |
| M4.Did you lose your balance when bending over to or reaching for something? | 1 | 2 | 3 | 4 | 5 |
| M6.Did you have trouble climbing stairs? | 1 | 2 | 3 | 4 | 5 |
| M7.Did you have trouble with needing to stop and rest when walking or using a wheelchair? | 1 | 2 | 3 | 4 | 5 |
| M8.Did you have trouble with standing? | 1 | 2 | 3 | 4 | 5 |
| M9.Did you have trouble getting out of a chair? | 1 | 2 | 3 | 4 | 5 |
| W1.Did you have trouble doing daily work around the house? | 1 | 2 | 3 | 4 | 5 |
| W2.Did you have trouble finishing jobs that you started? | 1 | 2 | 3 | 4 | 5 |
| W3.Did you have trouble doing the work you used to do? | 1 | 2 | 3 | 4 | 5 |
| UE1.Did you have trouble writing or typing? | 1 | 2 | 3 | 4 | 5 |
| UE2.Did you have trouble putting on socks? | 1 | 2 | 3 | 4 | 5 |
| UE3.Did you have trouble buttoning buttons? | 1 | 2 | 3 | 4 | 5 |
| UE4.Did you have trouble zipping a zipper? | 1 | 2 | 3 | 4 | 5 |
| UE5.Did you have trouble opening a jar? | 1 | 2 | 3 | 4 | 5 |

The next questions ask how much you agree or disagree with each statement because of the stroke. Each question deals with problem or feeling that some people have after a stroke. Circle the number in the box that best describes how you were doing during the past week.

**DURING THE LAST WEEK**:

|  | Strongly agree  1 | Moderately agree  2 | Neither agree nor disagree  3 | Moderately disagree  4 | Strongly disagree  5 |  |
| --- | --- | --- | --- | --- | --- | --- |
| T2.It was hard for me to concentrate. | 1 | 2 | 3 | 4 | 5 |  |
| T3.I had trouble remembering things. | 1 | 2 | 3 | 4 | 5 |  |
| T4.I had to write things down to remember them. | 1 | 2 | 3 | 4 | 5 |  |
| P1.I was irritable | 1 | 2 | 3 | 4 | 5 |  |
| P2.I was impatient with others | 1 | 2 | 3 | 4 | 5 |  |
| P3.My personality has changed | 1 | 2 | 3 | 4 | 5 |  |
| MD2.I was discouraged about my future. | 1 | 2 | 3 | 4 | 5 |  |
| MD3.I wasn’t interested in other people or activities | 1 | 2 | 3 | 4 | 5 |  |
| FR5.I didn’t join in activities just-for-fun with my family. | 1 | 2 | 3 | 4 | 5 |  |
| FR7.I felt I was a burden to my family | 1 | 2 | 3 | 4 | 5 |  |
| FR8.My physical condition interfered with my family life | 1 | 2 | 3 | 4 | 5 |  |
| SR1.Ididn‘t go out as often as I would like. | 1 | 2 | 3 | 4 | 5 |  |
| SR4.I did my hobbies and recreation for shorter period of time than I would like. | 1 | 2 | 3 | 4 | 5 |  |
| SR5.I didn’t see as many of my friends as I would like | 1 | 2 | 3 | 4 | 5 |  |
| SR6.I had sex less often than I would like | 1 | 2 | 3 | 4 | 5 |  |
| SR7.My physical condition interfered with my social life | 1 | 2 | 3 | 4 | 5 |  |
| MD6.I felt withdrawn from other people | 1 | 2 | 3 | 4 | 5 |  |
| MD7.I had little confidence in myself | 1 | 2 | 3 | 4 | 5 |  |
| MD8.I was not interested in food. | 1 | 2 | 3 | 4 | 5 |  |
| E1.I felt tired most of the time | 1 | 2 | 3 | 4 | 5 |  |
| E2.I had to stop and rest often during the day | 1 | 2 | 3 | 4 | 5 |  |
| E3.I was too tired to do what I wanted to do | 1 | 2 | 3 | 4 | 5 |  |

Now we would like you to say how you think you are doing today in some general areas compared to how you were **before your stroke.** Put an ‘X’ in the box to show whether each area is a lot worse, a little worse or the same as **before** your stroke. Please remember to compare how you are doing today with how you were **before the stroke happened.**

|  | A lot worse than before I had stoke | Somewhat worse than before I had stroke | A little worse than before I had stroke | The same as before I had stroke |
| --- | --- | --- | --- | --- |
| 1E. My energy level is |  |  |  |  |
| 2FR. My role in the family is |  |  |  |  |
| 3L. My language is |  |  |  |  |
| 4M. My mobility is |  |  |  |  |
| 5MD. My Mood is |  |  |  |  |
| 6P. My personality is |  |  |  |  |
| 7SC. I can take care of myself |  |  |  |  |
| 8SR. My role in the society is |  |  |  |  |
| 9T. My thinking is |  |  |  |  |
| 10UE. The use of my arm and  hand is |  |  |  |  |
| 11V. My vision |  |  |  |  |
| 12W. I do my jobs at home  or at work |  |  |  |  |
| 13.Overall my quality of life is |  |  |  |  |

**FINAL HAUSA VERSION OF Stroke Specific Quality of Life 2.0**

**SHANYEWAR BARIN JIKI: Tambayoyi a kan Inganta Hanyoyin Gudanar da rayuwa. Kashi na 2.0**

**UMARNI**

Wadannan jerin tambayoyi ne da akayi don bincike da sanin halin da kuke ciki dangane da ayyukanku na yau da kullum da kuma yadda kuke ji a jikinku, wanda a wasu lokuta matsalar shanyewar jiki kan iya shafa. Ga kowace tambaya ana bukatar ka bayyana yadda kaji ko yaddaka gudanar da ayyukanka suka kasance a makon da yawuce.

Kashin farko na tambayoyin, ana bukatar sanin irin yanayin matsalar da kukan ci karo da su yayin gudanar da wasu ayyuka kebantattu. Kowace tambaya ta tabo wasu matsaloli da mutane kan fuskanta bayan samun matsalar shanyewar barin jikinsu. Ka zagaye amsa ko zabin da kake ganin ya fi dacewa da irin matsalar da ka fuskanta yayin gudanar da wasu ayyuka, a satin da yagabata.

**A SATIN DA YA GABATA**

|  | **Ban iyayi sam-sam**  **1** | **Da matukar wahala**  **2** | **Da kyar**  **3** | **Da iyar matsala kadan**  **4** | **Ban sami**  **wata matsala**  **ba sam-sam**  **5** |  |
| --- | --- | --- | --- | --- | --- | --- |
| **SC1.** Kun samu matsala wajen girka abinci, ko zuwa sayen kayan abinci? |  |  |  |  |  |  |
| **SC2.** Ka samu matsala wajen tauna ko hadiyar abinci? |  |  |  |  |  |  |
| **SC4**. Ka samu matsala wajen sa kaya? Kamar sa safa ko takalmi ko sa maballi da jan zif na riga/da mazagi na wando, ko daura zani, ko kalabi, ko hijabi? |  |  |  |  |  |  |
| **SC5**. Ka samu matsala wajen yin wanka? |  |  |  |  |  |  |
| **SC8**. Ka samu matsala wajen yin amfani da bandaki? |  |  |  |  |  |  |
| **V1**. Ka samu cikas wajen jin dadin kallon talabijin kamar yadda ya kamata? |  |  |  |  |  |  |
| **V2**. Ka samu matsalar rashin iya kai wa ga wasu abubuwa saboda rashin karfin gani? |  |  |  |  |  |  |
| **V3**. Ka samu matsalar na gani da gefe guda na idanun ka? |  |  |  |  |  |  |
| **L2**. Ka samu cikas na yin magana? Kamar sarkewar harshe ko in’ina yayin magana? |  |  |  |  |  |  |
| L3. Ka sami matsalar yin Magana yadda ya kamata? Musamman wajen yin amfani da wayar hannu? |  |  |  |  |  |  |
| L5. Shin mutane sun sami matsalar fahimtar maganarka? |  |  |  |  |  |  |
| L6.Ka samu matsalar laluben Kalmar da za ka furta? |  |  |  |  |  |  |
| L7. Shin sai ka maimaita Magana sannan ake iya fahim tarka? |  |  |  |  |  |  |
| M1. Ka samu cikas wajen yin tafiya? (idan ba ka iya tafiya to ka kewaye tambaya M1 sannan ka tafi tambaya ta M7) |  |  |  |  |  |  |
| M4. Ka yi tangadi (taga-taga) yayin sunkuyawa ko wajen kokarin kaiwa ga wani abu? |  |  |  |  |  |  |
| M6. Ka samu matsalar hawa matattakalar bene? |  |  |  |  |  |  |
| M7. Ka samu matsalar bukatar neman tsayawa ko hutawa yayin tafiya ko wajen amfani da kujerar guragu? |  |  |  |  |  |  |
| M8. Ka samu matsala wajen tsayawa? |  |  |  |  |  |  |
| M9. Ka samu matsalar iya mikewa daga kan kujera? |  |  |  |  |  |  |
| W1. Ka samu matsala wajen yin ayyukan yau da kullum na gida? |  |  |  |  |  |  |
| W2. Ka samu matsalar kasa karasa ayyukan da ka fara? |  |  |  |  |  |  |
| W3. Ka samu matsalar gudanar da ayyukan da ka saba yi? |  |  |  |  |  |  |
| UE1. Ka samu matsalar yin rubutu da hannu ko da naura mai kokoiwa? |  |  |  |  |  |  |
| UE2. Ka samu matsala wajen saka safa? |  |  |  |  |  |  |
| UE3. Ka samu matsalar saka maballin riga? |  |  |  |  |  |  |
| UE4. Ka samu matsalar jan zif?  **UE5.** Ka samu matsala wajen bude murfin kwalba? |  |  |  |  |  |  |

Tambayoyi na gaba suna bukatar sanin matsayin yarda ko rashin yarda da bayanan da aka fada game da shanyewar barin jiki. Kowace tambaya ta shafi wata matsala ko wani yanayi da wasu mutane suka ji bayan sun sami matsalar shanyewar barin jiki. Ka zagaye tambaya da tafi dacewa da bayanin yadda ka sami kanka a makon da ya gabata.

A SATIN DA YA GABATA

|  | **Na yarda kwarai**  **1** | **Na yarda sama-sama**  **2** | **Ba ni da zabi**  **3** | **Ban yarda ba**  **4** | **Ban yarda ba sam-sam**  **5** |
| --- | --- | --- | --- | --- | --- |
| **T2.** Samun nutsuwa ya yi min wuya sosai |  |  |  |  |  |
| **T3.** Na samu matsalar tuna abubuwa |  |  |  |  |  |
| **T4**. Sai na rubuta abubuwa domin kadana manta su |  |  |  |  |  |
| **P1.** Na kasance mai saurin fushi |  |  |  |  |  |
| **P2.** Ba na iya hakuri (jure wa) da mutane |  |  |  |  |  |
| **P3.** Yanayina ya sauya |  |  |  |  |  |
| **MD2.** Banajin dadin rayuwata |  |  |  |  |  |
| **MD3.** Ban samu sha’awar shiga harkokin mutane ko wasu ayyuka ba |  |  |  |  |  |
| **FR5.** Ban shawar shiga sha’anonin mutane |  |  |  |  |  |
| **FR7**. Na ji kamar na zame wa iyalina wata matsala |  |  |  |  |  |
| **FR8.** Yanayin da na shiga ya kawo cikas a rayuwata da iyalina |  |  |  |  |  |
| **SR1**. Ban samu fita kamar yadda niske so ba |  |  |  |  |  |
| **SR4.** Inayin wasannina da nikeyi ba kamar yadda na sababa. |  |  |  |  |  |
| **SR5**. Ban samu ganin mafiyawan abokaina kamar yadda nake so. |  |  |  |  |  |
| **SR6.** Ban samu kusantar iyalina ba (jima’i) kamar yadda nake so. |  |  |  |  |  |
| **SR7.** Yanayin jikina ya kawo tarnaki wajen gudanar da rayuwata a cikin jama’a. |  |  |  |  |  |
| **MD6.** Na ji kamar na yi nesa da mutane. |  |  |  |  |  |
| **MD7.** Na karaya da kaina. |  |  |  |  |  |
| **MD8.** Ban jisha’awar cin abinci ba. |  |  |  |  |  |
| **E1**. Na ji gajiya a mafi yawan lokaci. |  |  |  |  |  |
| **E2**. Da rana, ina tsayawa na huta yayin tafiya. |  |  |  |  |  |
| **E3**. Saboda gajiya sosai ba na iya yin abin da nake son yi. |  |  |  |  |  |

Yanzu kuma muna bukatar ka fada mana yadda ka sami kanka yanzu a wasu bangarori, idan aka kwatanta da yadda kake ji kafin wannan matsala ta same ka. Ka sa alamar ‘X’ a gurbin da ya dace. Ko dai abin ya yi muni sosai ko ya yi muni kadan ko yana nan kamar yadda yake da kafin shanyewar barin jiki. Ka tuna dai, ka dinga gwada yadda kake ji yanzu da kuma yadda kakeji kafin ka samu rashin lafiyar.

|  | (yay i) muni sosai kamin na samu rashin lafiyar  1 | (yayi) muni kamin na samu rashin lafiyar  2 | (yayi) muni kadan kamin na samu rashin lafiyar  3 | Daida iyake  da kamin na  samu rashin  lafiyar  4 |
| --- | --- | --- | --- | --- |
| **1E** Kuzarina |  |  |  |  |
| **2FR** Matsayina a cikin iyali |  |  |  |  |
| **3L.** Harshena/Maganata |  |  |  |  |
| **4M.** Zirga-zirgata |  |  |  |  |
| **5MD.** Halin zuciyata/yanayina |  |  |  |  |
| **6P.** Kamalata |  |  |  |  |
| **7SC.** Zan iya kula da kaina |  |  |  |  |
| **8SR**. Matsayina a cikin al’umma |  |  |  |  |
| **9T.** Tunanina |  |  |  |  |
| **10UE.** Yin amfani da hannayena |  |  |  |  |
| **11V.** Ganina |  |  |  |  |
| **12W.** Ina yin ayyukana a gida ko wajen aiki/sana’a |  |  |  |  |
| 13 – Rayuwata gaba daya |  |  |  |  |

HAUSA TRANSLATION OF WHOQOL-BREF

Ka/Ki karanta kowaccc tambaya’ Ka/Ki auna damuwar ka/ki, zagayc lambar amsar da tafi daccwa.

| Yaya kake ganin | Talaka tilis Talaka | Ba talaka ba, ba mai kudi ba | Da kyau | Da kyan kwarai | Matsayin rayuwarka |
| --- | --- | --- | --- | --- | --- |
|  | 1 | 2 | 3 | 4 | 5 |

| Ya ya kwanciyar hankalinka a kan lafiyar jikinka | Bangamsu ba kwata kwata | Bangamsu ba | Ba yabo, ba fallasa | Na gamsu | Na gamsu sosai |
| --- | --- | --- | --- | --- | --- |
|  | 1 | 2 | 3 | 4 | 5 |

Wadannan tambayoyin suna Magana na kan yadda wadansu abubuwa suka shafe ka mokonni biyu da suke shude.

| Zafin ciwo na iy a hana ka aikatu abin da ke so? | Sam bai hana ni | Yakan dan hani | Zafi daidai gwargwando | Zafi sosai | Matsananci |
| --- | --- | --- | --- | --- | --- |
|  | 1 | 2 | 3 | 4 | 5 |

| Kana bukatar shan magani karfin ka iya gudanar da rayuwarka | Bani bukata | Dan kadan | Daidai gwargwado | Sosai | Da yawa kwarai |
| --- | --- | --- | --- | --- | --- |
|  | 1 | 2 | 3 | 4 | 5 |

| Ta yaya ke jin Raynwarka na da anfani | Babu anfani | Kadan | Daidai gwargwado | Sosai | Matuka |
| --- | --- | --- | --- | --- | --- |
|  | 1 | 2 | 3 | 4 | 5 |

| Kana samun natsuwa sosai? | Ba ni samu | Kadan | Daidai gwargwando | Sosai | Mutuka |
| --- | --- | --- | --- | --- | --- |
|  | 1 | 2 | 3 | 4 | 5 |

| Ta yaya kake jin rayuwar bat a cikin hadari | Ba ta ciki | Kadan | Daidai gwargwando | Sosai | Mutuka |
| --- | --- | --- | --- | --- | --- |
|  | 1 | 2 | 3 | 4 | 5 |

| Mahalinka na cikin koshin lafiya | Ba bu | Kadan | Daidai gwargwando | Sosai | Mutuka |
| --- | --- | --- | --- | --- | --- |
|  | 1 | 2 | 3 | 4 | 5 |

Wadannan tambayoyi da ke biye na bineike ne a kan yadda kake yi ko jin wasu abubuwa a makonni biyu da suka shude.

| Kana da cikakke kartin gudannar da rayuwarka? | Ba ni da shi | Kadan | Daidai gwargwando | Sosai | Mutuka |
| --- | --- | --- | --- | --- | --- |
|  | 1 | 2 | 3 | 4 | 5 |

| Ka yi na’am da yadda jikinka yake? | Ban yi ba | Kadan | Daidai gwargwando | Sosai | Mutuka |
| --- | --- | --- | --- | --- | --- |
|  | 1 | 2 | 3 | 4 | 5 |

| Kana da cikakken kudiu godanar da rayuwarka | Ba ni da shi | Kadan | Daidai gwargwando | Sosai | Mutuka |
| --- | --- | --- | --- | --- | --- |
|  | 1 | 2 | 3 | 4 | 5 |

| Kana samun bayauia da kake hukata a rayuwarka a kai a kai? | Ba ni samu | Kadan | Daidai gwargwando | Sosai | Mutuka |
| --- | --- | --- | --- | --- | --- |
|  | 1 | 2 | 3 | 4 | 5 |

| Kana samun lokacin shkatawa |  |  |  |  |  |
| --- | --- | --- | --- | --- | --- |
|  | 1 | 2 | 3 | 4 | 5 |

| Kana iya zuwa ko’ina? | Dad wuya sosai | Da wuya | Ba yabo ba fallasa | Sosai | Matuka |
| --- | --- | --- | --- | --- | --- |
|  | 1 | 2 | 3 | 4 | 5 |

Wadannan tambayoyi da ke biye ne bukata ne ka bayyana yadda ka gamsu da abubuwa da dama da suka shafi rayuwarka a makonni biyu da surka shude.

|  |  | Ban gamsu bas am sam. | Ban gamsu ba. | Ba yabo ba fallasa | Na gamsu | Na gamsu matuka |
| --- | --- | --- | --- | --- | --- | --- |
| 16(f.3) | Ka gamsu da yadda kake samun barei? | 1 | 2 | 3 | 4 | 5 |
| 17(f10.3) | Ka gamsu da yadda kake samur aikata abubuwan da. | 1 | 2 | 3 | 4 | 5 |
| 18(f12.4) | Ka gamsu da yawan aikin da kake yi? | 1 | 2 | 3 | 4 | 5 |
| 19(f6.3) | Ka gamsu da kanka? | 1 | 2 | 3 | 4 | 5 |
| 20(E13.3) | Ka gamsu da abokan huldarka? | 1 | 2 | 3 | 4 | 5 |
| 21(F15.3) | Ka gamsu da yadda kake kwanciya da abokan zama. | 1 | 2 | 3 | 4 | 5 |
| 22(F14.4) | Ka gamsu dad a irin aimakon da kake samu daga abokanka? | 1 | 2 | 3 | 4 | 5 |
| 23(F17.3) | Ka gamsu da yanayin muhallinka? | 1 | 2 | 3 | 4 | 5 |
| 24(F19.3) | Ka gamsu da yadda kake samun abubuwan kula da lafiyarka. | 1 | 2 | 3 | 4 | 5 |
| 25(F23.3) | Ka gamsu da hanyar sufurinka? | 1 | 2 | 3 | 4 | 5 |

Tambayar da ke biye na Magana ne a kan kake ji ko gagin abubuwa da suka shafi rayuwarka.

| 26(F8.1) |  | Sam ba ni samu | Ba koyaushe ba | A kai a kai | Da yawa sosai | A koyaushe |
| --- | --- | --- | --- | --- | --- | --- |
|  | Kana yawan samun damuwa kon faduwar gaba ko ka jiba ka kaunar rayuwa? | 1 | 2 | 3 | 4 | 5 |
